# Ovarian cancer initially presenting with isolated ipsilateral superficial inguinal lymph node metastasis: a case study and review of the literature

**DOI:** 10.1186/1757-2215-7-20

**Published:** 2014-02-10

**Authors:** Xiao-Jun Yang, Fei-Yun Zheng, Yun-Sheng Xu, Rong-Ying Ou

**Affiliations:** 1 Department of Obstetrics and Gynecology, The First Affiliated Hospital of Wenzhou Medical University, Wenzhou 325000, China; 2 Department of Obstetrics and Gynecology, The First Affiliated Hospital of Soochow University, Suzhou 215006, China; 3 Department of dermatology, The First Affiliated Hospital of Wenzhou Medical University, Wenzhou 325000, China

**Keywords:** Ovarian cancer, Superficial inguinal lymph node, Lymphatic spread

## Abstract

Isolated superficial inguinal metastases without any extended intra-abdominal spread is a rare event in patients with ovarian carcinoma. Here we report an isolated superficial inguinal metastasis in a patient with primary ovarian cancer. A 54-year-old Chinese patient with primary ovarian cancer, had an isolated painless enlarged right groin swelling (3×2cm) as the only manifestation, preoperative pathology confirmed metastatic adenocarcinoma. Gynecologic examination, transvaginal ultrasonography of the abdominopelvic cavity revealed a 5-cm mixed, right adnexal mass. At exploratory laparotomy, there was little intra-abdominal tumor dissemination but 100 ml of faint yellow peritoneal fluid and a 5-cm right ovarian tumor with intact capsule. Staging operation was performed and postoperative pathology confirmed adenocarcinoma located within right ovarian, with no evidence of involvement of other sites. Then the patient received adjuvant chemotherapy for Stage IVB. Five years later, the patient is currently still alive without evidence of recurrent disease. This case indicate that ovarian carcinoma isn’t a disease localized only within the intra-peritoneal cavity, isolated superficial inguinal lymph node metastasis might occur in rare cases via potential lymphatic and (or) hematogenous route under special conditions. We propose the need to investigate the possible mechanisms, risk factors, metastatic patterns, the biology and natural history of such patients in a large-scale and multicenter analysis. Furthermore, efforts should be made for earlier and differential diagnosis and finally prolong survival time for such patients.

## Background

Ovarian carcinoma is the most frequent cause of death from gynecological malignancies in China [1]. The main reason of its high mortality is due to the lack of symptoms for early detecting. Over 70% of patients with ovarian carcinoma were diagnosed as International Federation of Gynecology and Obstetrics (FIGO) stage III or IV at their initial presentation [2]. The most frequent symptoms include abdominal pain, distension, early satiety, vaginal bleeding or a combination of these, and the most common sign present at initial visit is a pelvic mass [3]. Patients with ovarian cancer were reported to presented with distant metastatic deposits in the cervix, vagina, or vulva at their initial visit [4]. Lymph node metastasis occurred in about 14-70% of patients with ovarian carcinoma and distributed mainly in the pelvic and aortic region [5]. Nevertheless, it is uncommon to present superficial inguinal lymph node (SILN) metastasis in patients with early stage of ovarian carcinoma. Isolated SILN metastasis was a very rare event in patients with ovarian carcinoma [6]. Here we report a 54-year-old patient with complete clinicopathological data, who attacked by occult primary ovarian cancer limited within the right ovary, while initially presented with an asymptomatic isolated enlarged right SILN and confirmed to be a metastatic adenocarcinoma by preoperative pathological examination. Its potential implications in basic science research and clinical management are discussed.

## Case description

**Figure 1:**
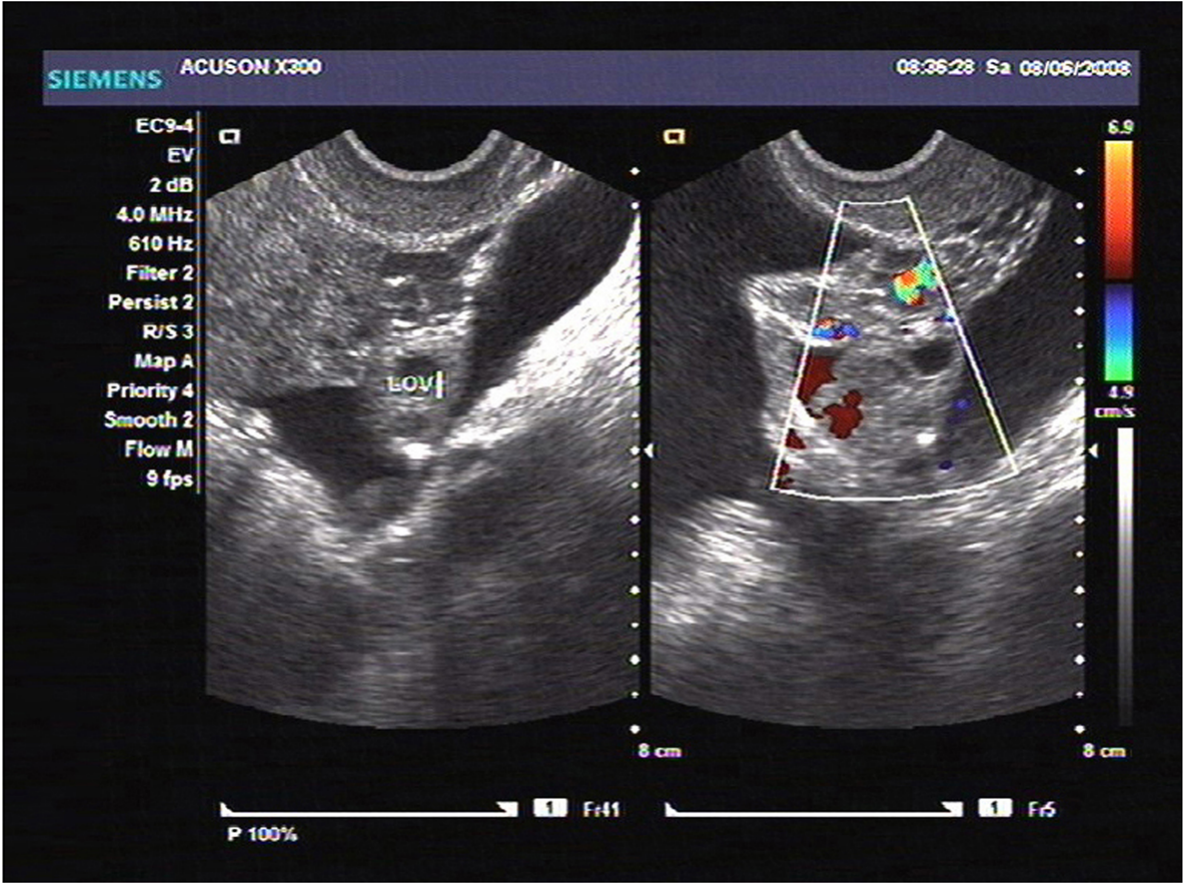
**Preoperative transvaginal ultrasonography (TVUSG) showed a 5 × 4 × 5 cm mixed lump within the right adnexa.** It has relatively rich blood supply signals on the circumference and inside tumor, with papillary vegetation and irregular septa, together with a small amount of pelvic fluid.

**Figure 2:**
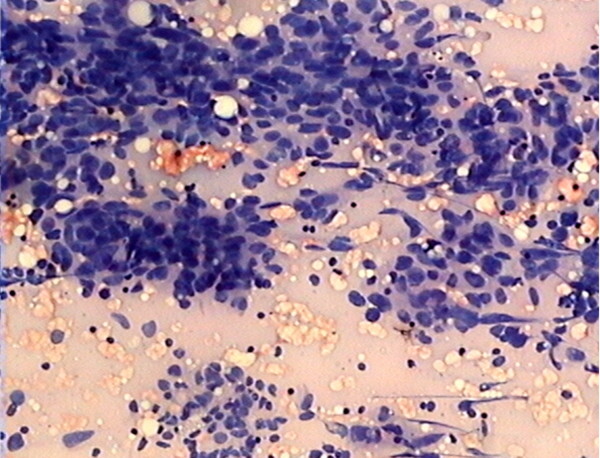
Preoperative pathologic diagnosis by fine needle aspiration demonstrated a metastatic adenocarcinoma within right SILN (haematoxylin and eosin stain; original magnification, ×50).

In June 2008, a 54-year-old Chinese woman, postmenopausal for 8 years, presented to our hospital with complaints of an isolated painless enlarged mass at right groin. On gross inspection, a palpable painless enlarged subcutaneous swelling (3 × 2 cm) were observed within the right groin. The contralateral inguinal nodes and the scalene nodes were clinically negative. Gynecologic examination showed a 5 cm fixed mass within the right adnexa. Transvaginal ultrasonography (TVUSG) showed a 5 × 4 × 5 cm mixed lump within the right adnexa, has relatively rich blood supply signals on the circumference and inside tumor, with papillary vegetation and irregular septa, together with a small amount of pelvic fluid (Figure 1). Results of endometrial and cervical biopsies, thyroid sonography, gastroduodenoscopy, colonoscopy, were all negative. Serum tumor markers including CA125 were all within normal range. Systematic infectious disease that can cause enlarged inguinal lymph node were analyzed, including hepatitis A, hepatitis B, hepatitis C, syphilis, HIV, HSV, and the results were all negative. No ulcers were presented in the lower genital tract. Patient was also screen for potential presence of Trichomonas vaginalis, genital Chlamydia trachomatis, and Neisseria gonorrhoeae, and results were all negative. Five days later, the patient received fine needle aspiration for the right groin swelling and preoperative pathological examination confirmed a metastatic adenocarcinoma (Figure 2).

Eleven days later, exploratory laparotomy were performed, there was little intra-abdominal tumor dissemination but 100 ml of faint yellow peritoneal fluid and a 5-cm right ovarian tumor with intact capsule, the rest of abdominopelvic cavity remained macroscopically negative. During the surgery, frozen sections for right adnexal mass revealed a low-grade differentiated serous ovarian papilliferous cystadenocarcinoma. According to procedures of staging operation for ovarian carcinoma [2], total abdominal hysterectomy, bilateral salpingo-ovariectomy, complete removal of the omentum, appendectomy, random biopsies of the peritoneum, systematic pelvic and paraaortic lymphadenectomy were performed, completed with excision of the enlarged node in the right groin.

**Figure 3:**
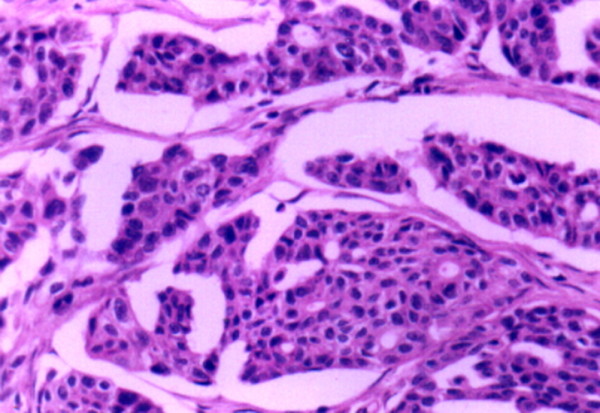
**Postoperative pathologic diagnosis confirmed poorly differentiated serous papilliferous cystadenocarcinoma of the right ovary (FIGO G3).** Cancer cells were arranged to form irregular solid and reticular structures. (haematoxylin and eosin stain; original magnification, ×50).

**Figure 4:**
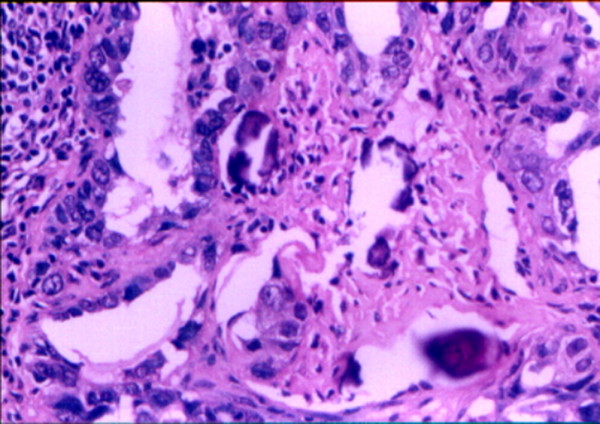
**Postoperative pathologic diagnosis show poorly differentiated serous cystadenocarcinoma within SILN metastases from ovarian cancer.** There were psammoma bodies in tumor stroma, consistent with preoperative fine needle aspiration.(haematoxylin and eosin stain; original magnification, ×100).

**Figure 5:**
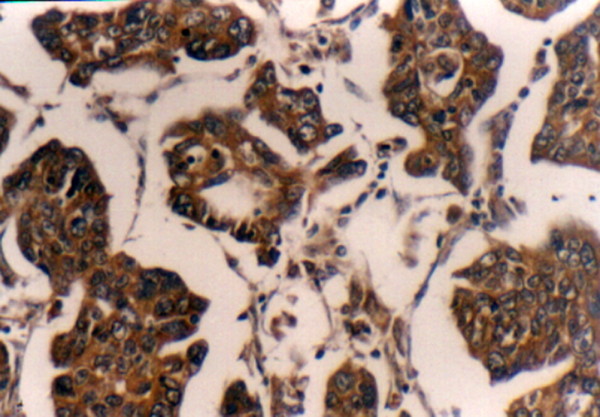
Postoperative positive cytoplasmic immunostaining of CA125 was detected in primary ovarian cancer tissues (CA125 receptor stain; original magnification: ×100).

**Figure 6:**
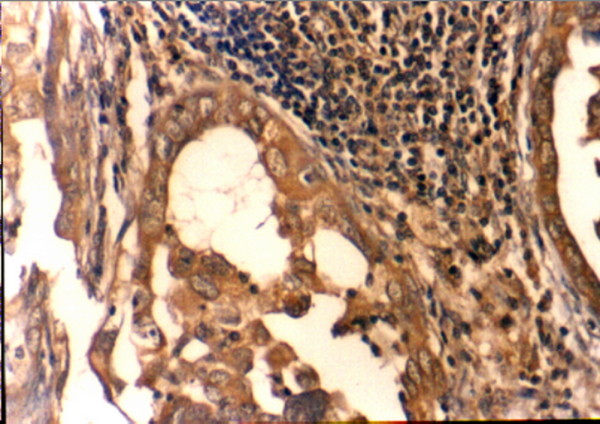
Postoperative positive cytoplasmic immunostaining of CA125 was detected in SILN metastases from ovarian cancer (CA125 receptor stain; original magnification: ×100).

**Figure 7:**
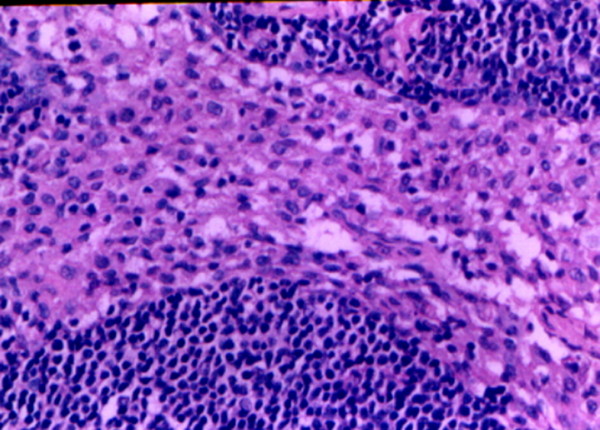
**Histological features of non-metastatic pelvic and paraaortic lymph nodes from ovarian cancer (haematoxylin and eosin stain; original magnification: ×100).** Present with lymphonode reactive hyperplasia and without tumor cells invasion.

Postoperative pathologic diagnosis showed a poorly differentiated serous papilliferous cystadenocarcinoma of the right ovary (Figure 3), and right inguinal lymph node metastasis (Figure 4), which consistent with preoperative fine needle aspiration, immunohistochemical staining showed positive cytoplasmic CA125 expression both in ovarian cancer tissues (Figure 5) and in metastatic SILN (Figure 6), cytology of the pelvic fluid showed poor differentiated adenocarcinoma cells (FIGO G3). The rest of pathological diagnosis showed no evidence of disseminated intraperitoneal and retroperitoneal metastatic disease, all pelvic and paraaortic lymph nodes were negative (Figure 7). The patient was diagnosed to be FIGO Stage IVB [7] and then referred to postoperative adjuvant chemotherapy with paclitaxel (175 mg/m^2^) and carboplatin (AUC-5) at 28-day intervals for six cycles. Chemotherapy was completed in January 2009. Presently, five years after the primary diagnosis, this patient is still alive with no evidence of recurrent disease.

## Discussion

### Incidence of inguinal lymph node metastasis

Lymph node metastases can be well recognized in ovarian cancer with sampling of retroperitoneal lymph nodes as an integral part of the staging operation [8]. FIGO introduced inguinal lymph node involvement into the definition of stage IVB in ovarian carcinoma since 2013 [7], while patients exhibited metastatic retroperitoneal lymph nodes are classified as stage IIIC even when the primary tumor is limited to the pelvis [9]. The most common way of spreading in ovarian carcinoma is lymphatic metastasis and transcoelomic spread to adjacent viscera, with distant metastasis often concurring with extensive intra-abdominal dissemination [10]. Nevertheless, isolated SILN involvement in patients without any extended intraabdominal spread is a rare event [6,11,12].

The incidence of inguinal lymph node metastasis in ovarian carcinoma was approximately 3-5% as reported in literatures [6,11,13,14], while these studies can't provide enough clinical informations and pathological features. A little bit more detailed descriptions include following cases. A patient presenting with an enlarged inguinal lymph node, was finally diagnosed to be endometrioid carcinoma of the ovary [15]; a patient presented with metastatic inguinal lymphadenopathy was confirmed with poorly differentiated ovarian malignancy until 33 months later [16]; an eighteen-year old female patient complained with enlarged inguinal lymph nodes and secondary lymphedema of both legs, showed ovarian tumors (approximately 6 cm diameter) detected by computed tomography, and was finally proved to be stromal infiltration of signet-ring cell carcinoma in both ovaries [17]; a 48-year-old patient presented with 6-month history of inguinal swelling, was confirmed with a serous papillary ovarian carcinoma, thus indicated that inguinal lymphadenopathy can be initial symptom epithelial ovarian carcinoma [18]. Nevertheless, these studies were also inconclusive, since they neglected to give any further detailed information on pathological examination of the pelvic and paraaortic lymph nodes, and whether inguinal lymph node metastasis occurred in isolation or concurred with other sites of neoplasm metastasis.

To the best of our knowledge, the following three records provided full clinicopathological features in this regards. Scholz et al. [19] firstly reported a patient with undifferentiated serous adenocarcinoma of both ovaries (10 × 5.5 cm), and involvement of fimbria of the right fallopian tube, and positive peritoneal washing, initially displayed an isolated left inguinal node metastasis, without other nodal groups involvement. Then Manci et al. [20] reported that, a patient complained with bilateral inguinal lymphadenopathy, showed an increased uptake of fluorodeoxyglucose in the inguinal and both adnexal areas as detected by [(18)F] fluorodeoxyglucose (FDG) positron emission tomography (PET), then post-operational pathological diagnosis confirmed low-grade differentiation serous papilliferous adenocarcinoma of both ovaries (size not known), and metastatic bilateral inguinal lymph nodes, without any intraperitoneal or lymphatic spread. Afterwards, Ang et al. [21] reported a patient with left ovarian adenocarcinoma (9.0 × 6.4 cm) presented with isolated metastasis to the right inguinal lymph node, there were no other sites of involvement. Thus, the case we reported was one of the few cases which have complete clinicopathological informations in existing literatures. However, different from Scholz’s and Ang's case, in which large (10 × 5.5 cm) and (9.0 × 6.4 cm) tumor burden were found, our case demonstrated relatively small tumor burden (5 cm in largest diameter), disease was localized only within the right ovary, initially presented with right SILN metastasis, without any evidence of extensive intra-abdominal dissemination, and retroperitoneal pelvic or paraaortic lymph nodes metastasis. Therefore, this is the fourth case of ovarian carcinoma which presented with isolated SILN metastasis reported in existing literatures.

### Diagnosis and prognosis

Ovarian carcinoma usually presents with advanced stage at their initial visit (FIGO Stage III and IV), with signs and symptoms related to the diffused intraperitoneal disease [2]. However, the presence of asymptomatic isolated SILN at the time of first visit frequently creates a diagnostic dilemma. This situation is unique not only in the manner of disease presentation, but also in the time lag from first seeking advice to evidence of intra-abdominal malignancy [16].

In general, the common condition which might presented with palpable SILN enlargement include metastatic disease or secondary inflammation [22]. Pathology of tumors commonly metastasising to the inguinal lymph nodes include breast cancer [23]; tumours arising from the vulva and lower third of the vagina [24]; pelvic malignancies [25]; malignant tumours of the skin, most commonly primary malignant melanoma or squamous cell carcinoma arising on the legs and trunk [14,24]; squamous cell carcinoma of the anal canal is also a common gastrointestinal tumour to metastasise to the inguinal lymph nodes [24]. Systematic infectious disease such as syphilis, HIV, HSV, and local infection such as ulcers in the lower genital tract, Neisseria gonorrhoeae, can also cause enlarged inguinal lymph node. Therefore, preoperative diagnosis of inguinal lymph node enlargement always cause diagnostic dilemma and might involve general practitioners, oncologists, dermatologists, and specialist nurses. However, antibiotics treatment for 4 to 6 week is usually prescribed, followed by re-evaluation of the lymphadenopathy [24]. Previous literatures showed that, for patients with isolated inguinal metastasis of unknown origin, laparoscopic surgery provided a minimally invasive diagnostic approach of the abdominal and pelvic cavity, although there controversy that a small tumor within the ovary might be missed [22]. Imaging examination such as TVUSG maybe helpful in detecting the earlier malignancy in ovary. PET might has an appropriate role in the diagnosis of occult ovarian neoplasm, even in the absence of a CA125 elevation [20]. The combination of FDG-PET/CT was successfully used to identify ovarian cancer recurrence in an inguinal hernia sac [26]. Moreover, serum levels of tumor markers such as CA125 can also assist to determine the primary disease when the clinical presentation is atypical or confusing. However, in our case, we proposed that, the management of a patient presenting with inguinal enlargement of unknown origin should include at least a detailed case history collection, complete gynecological examination and some useful auxiliary diagnostic measures for any ovarian neoplasms. Sometimes, even if no evident clinical signs and symptoms of a tumor in the lower genital tract, isolated enlarged SILN should also be paid enough attention for possible existence of an occult malignant ovarian tumor. In other words, ovarian cancer should be part of the differential diagnosis in women with inguinal lymphadenopathy even without any clinical evidence of intra-abdominal disease. Furthermore, surgical excision or lymph node biopsy can be a indication for inguinal lymphadenectomy, can provide better diagnostic and prognostic information.

The role of lymph node metastasis on survival in ovarian cancer has been a matter of debate over the years [27]. Generally, lymph node metastasis is recognized as a parameter of unfavorable prognosis. The prognosis of distant metastasis in ovarian carcinoma is poor and the median survival was only about 12 months [28]. While the opposite view suggested that, patients with ovarian carcinoma which upstaged to stage III based solely on systematic lymphadenectomy, have similar survival to stage I/II patients and superior survival to other stage III patients [29]. In fact, survival difference between node-positive-only stage IIIC and intra-abdominal stage IIIC simply reflect the prognostic impact of small versus large tumor size [30]. “Node-positive-only” stage IIIC have a more favorable outcome than intra-abdominal stage IIIC and IIIA/B in patients with epithelial ovarian cancer [31,32].

The impact of SILN metastasis on the prognosis in ovarian cancer is also in controversy. Some authors suggested that patients with inguinal lymph node metastasis as their first symptoms were associated with poor prognosis, and can only survive for about three years [16,18]. While others argued that, in patients with recurrent epithelial carcinoma, who presented as isolated lymph node metastases (including inguinal nodal involvement), complete optimal secondary cytoreductive surgery was achievable in the majority of cases and were associated with relatively favorable long-term survival outcome [33,34]. Similar study also indicated that, for those suffered with serous carcinoma of the ovary, fallopian tube, or peritoneum, distant lymph node metastasis was an uncommon event (including inguinal nodal involvement), however, this rare presentation does not adversely affect survival, patients with minimal intra-peritoneal disease and extra-abdominal lymph node metastases survive longer than those with bulky peritoneal disease [35]. According to the new stage system, inguinal lymph node metastasis was classified into FIGO Stage IVB ovarian cancer [7]. While in the previous edition of stage system, it was confusion about this, and inguinal lymph node metastasis was usually put into Stage IIIc [9]. Therefore, we supposed that, such stage difference might cause inconsistency in data analysis on prognosis regarding inguinal lymph node metastasis.

The case reported here, was confirmed to be Stage IVB ovarian cancer, and survive for five years after six rounds of carboplatin plus paclitaxel systematic chemotherapy, with no evidence of recurrence. We consider it that such patients presented only with distant lymphatic metastasis, were in relatively better conditions and specific immune status, thus have better prognosis after comprehensive treatment, as compared with bulky peritoneal disease. However, with the issue of new stage system in the year of 2013 [7], large-scale and multicenter analysis should be done to investigate clinical outcome in patients with ovarian cancer confined to the ovary but upstaged to stage IVB due to metastatic SILN, and provide more insight about potential differences in biological and clinical behavior of inguinal lymph node versus intra-peritoneal metastasis. Furthermore, there is no existing guideline on definitive management of patients with ovarian cancers metastasizing to isolated SILN [22], efforts should be made to improve early diagnosis and finally prolong the survival of such patients. We suggested that, such patients should be entered into clinical trials of different treatment modalities in order to develop optimal clinical guideline.

### Routes and mechanisms of lymphatic metastasis

Generally, ovarian cancer has three routes for lymphatic metastasis [36,37]. Firstly, lymphatic vessels mainly accompany the ovarian vessels within the infundibulopelvic ligament towards the paraaortic and paracaval lymph nodes. Thus, nodes running parallel to lymphatic vessels are at highest risk of involvement. Once the pelvic and paraaortic lymph nodes have been involved, lymphatic channels within the diaphragm and retroperitoneum will facilitate dissemination above the diaphragm. Less commonly, the second route follows the subovarian plexus in the bilateral broad ligament towards the obturator and pelvic iliac lymph nodes. The third potential route follows the bilateral round ligament of the uterus to the external iliac and deep inguinal lymph nodes. We suggested that, in the absence of paraaortic or pelvic lymphadenopathy which mainly depend on the first and the second route as above mentioned, the isolated SILN metastasis might attribute to the third potential channel. Our case just provided support to lymphatic dissemination via this potential channel, through which ovarian cancer metastasize from the round ligament to deep inguinal lymph node and finally drained towards the SILN. However, the existence of such a potential pathway has not been confirmed yet. Moreover, hematogenous dissemination is also another possible pathway for this special metastatic pattern, and was reported to occur in approximately 2% to 3% of patients with primary ovarian carcinoma [10]. Theoretically, hematogenous route may account for dissemination to any distant sites in ovarian cancer. Such as, early extra-abdominal metastases [38], central nervous system metastases [39], axillary lymph nodes [40], and breast metastases [41,42], all support the model of spreading through hematogenous route. Therefore, in summary, we proposed that the event of isolated SILN metastasis in ovarian cancer potentially involved two channels: via deep inguinal lymphatic routes through round ligament and/or hematogenous route.

In addition to study the routes of isolated SILN metastasis in ovarian cancer, we should also explore its underlying mechanisms. Current study revealed that advanced tumor stage, low-grade cell differentiation were risk factors for the development of distant metastasis [28]. For instance, in our case with G3, low-grade cell differentiation might contribute to one of important risk factors for isolated SILN metastasis. In addition, mutation of p53 tumor suppressor gene was more likely to be associated with distant lymph node metastases in ovarian cancer, indicated that gene mutation and vascularization might also contribute to distant metastasis in ovarian cancer [43]. Moreover, we speculated that, this special metastatic pattern is probably the result of tumor biology and host-tumor immunostatus. The special host immune state within a specific time window perhaps plays a key role, might kill some primary cancer cells but neglect distant isolated lymph node metastasis. Nevertheless, there may be a number of other unknown factors beyond our present knowledge, such as unexplained hormones contributions. Existing literatures have put more emphasis on pelvic and paraaortic lymph nodes metastasis in ovarian cancer [36]. However, few studies had focused on the isolated SILN metastasis, the exact molecular mechanisms and/or risk factors of this special clinic metastatic pattern in ovarian cancer still deserve further investigation.

## Conclusions

Ovarian carcinoma isn’t a disease localized only within the intraperitoneal cavity, isolated SILN metastasis can occur and present as an initial symptom in rare case with relative initial tumor origin stage, via potential lymphatic and (or) hematogenous route under special mechanisms which might include roles of immunological, pathological and hormonal factors. On the other hand, it should be kept in mind that inguinal masses might be the metastatic lesions of ovarian cancer. A proper preoperative evaluation including gynecological exam, cervicovaginal smear, CA 125 level and TVUSG must be performed in such cases.

## Consent

Written informed consent was obtained from the patient for publication of this Case report and any accompanying images. A copy of the written consent is available for review by the Editor-in-Chief of this journal.

## Abbreviations

FIGO: International Federation of Gynecology and Obstetrics; FDG: Fluorodeoxyglucose; PET: Positron emission tomography; SILN: Superficial inguinal lymph node; TVUSG: Transvaginal ultrasonography.

## Competing interests

The authors declared that they have no competing interests, have no commercial, proprietary, or financial interest in the products or companies described in this article. X-J.Y. has nothing to disclose. F-Y. Z. has nothing to disclose. Y-S.X. has nothing to disclose. R-Y.O. has nothing to disclose.

## Authors’ contributions

X-J.Y. and R-Y.O. have contributed significantly in drafting the manuscript, literature review and revising it critically, F-Y.Z. provided the clinical data, Y-S.X. and R-Y.O. also involved in immunohistochemical staining of CA125, pathological diagnosis. All the authors had read and approved the final manuscript.
